# Motivational Interviewing in Pediatric Obesity: A Meta-analysis of the Effects on Behavioral Outcomes

**DOI:** 10.1093/abm/kaad006

**Published:** 2023-05-17

**Authors:** Tsui-Sui A Kao, Jiying Ling, Christina Vu, Rachel Hawn, Harrietta Christodoulos

**Affiliations:** College of Nursing, Michigan State University, USA; College of Nursing, Michigan State University, USA; College of Nursing, Michigan State University, USA; College of Nursing, Michigan State University, USA; College of Human Medicine, Michigan State University, USA

**Keywords:** Lifestyle modification, Eating, Physical activity, Snacks, Surgery beverages

## Abstract

**Background:**

Currently, the effects of motivational interviewing (MI) on children’s behavioral changes remain obscure.

**Purpose:**

This systematic review and meta-analysis examined the effects of MI on children’s lifestyle behavioral changes (fruits and vegetables [F/V], dairy, sugary beverages, calories, snacks, fat intake, moderate vigorous physical activity [MVPA], and screen time).

**Methods:**

Six databases (CINAHL, Cochrane, Embase, PsycINFO, PubMed, and Web of Sciences) from 2005 to 2022 were searched. Thirty-one intervention studies with a comparison group met the criteria. Random-effects models were performed to estimate the pooled effects; exploratory moderation analyses with mixed-effects models were used to explore potential intervention moderators.

**Results:**

The pooled effect size was 0.10 (*p* = .334) on ↑F/V, 0.02 (*p* = .724) on ↑dairy, −0.29 (*p* < .001) on ↓calories, −0.16 (*p* = .054) on ↓sugary beverages, −0.22 (*p* = .002) on ↓snacks, −0.20 (*p* = .044) on ↓fat, 0.22 (*p* = .001) on ↑MVPA, and −0.06 (*p* = .176) on ↓screen time. The effects of MIs were moderated by ↑MI sessions regarding ↓snacks (*B* = −0.04, *p* = .010). Multicomponent and clinical programs had greater effects on dairy intake than their counterparts (0.09 vs. −0.21, *p* = .034; 0.12 vs. −0.14, *p* = .027, respectively). Similarly, interventions with a fidelity check resulted in greater dairy intake than those without a check (0.29 vs. −0.15, *p* = .014). A few long-term follow-up assessments revealed effects on ↓F/V (−0.18; *p* = .143, *k* = 2), ↓dairy (−0.13, *p* = .399, *k* = 4), ↓MVPA (−0.04; *p* = .611, *k* = 6), and ↑screen time (0.12; *p* = .242, *k* = 4).

**Conclusions:**

Our findings support the short-term effects of MI on improving children’s lifestyle behaviors. Additional investigations are needed to better sustain children’s long-term behavioral changes.

## Teaser

Motivational interviewing shows short-term effects on improving children’s eating and physical activity behaviors. Interventions focusing on obesity treatment result in better effects than those targeting obesity prevention.

## Context

The purpose of this systematic review was to accomplish the following: (a) examine the effectiveness of motivational interviewing (MI) on improving children’s lifestyle behavioral changes (fruits and vegetables [F/V], dairy, sugary beverages, calorie, snacks, fat intake, moderate vigorous physical activity [MVPA], and screen time); (b) assess the potential intervention moderators (e.g., delivery format, duration, setting, children’s age, weight status, targeted behavior(s), parental involvement, and fidelity check); and (c) explore the long-term follow-up effects on available outcomes.

## Consequences of Poor Lifestyle Behaviors Among Children

Children’s poor eating habits (↓F/V, ↓dairy, ↑sugary beverages, ↑calorie, ↑snacks, and ↑fat intake) and poor physical activity (PA) patterns (↓MVPA and ↑screen time) are recognized as obesity-related behaviors and significantly contribute to childhood obesity [1–3]. Children with overweight or obese are at increased risk for chronic comorbidities in adulthood, including cardiovascular diseases, hypertension, and type 2 diabetes (T2DM) [4, 5]. Without effective behavioral interventions, children with poor lifestyle behaviors will be more likely to develop overweight or obese, and subsequently, be at an increased risk for many preventable health conditions. Empirical evidence suggests that combining healthy eating behaviors with the recommended amount of PA may help achieve healthier weights and reduce risk for chronic diseases [6]. However, programs that are successful in motivating and sustaining children’s lifestyles remain scarce.

*Motivational interviewing* is an evidence-based, person-centered counseling technique used to empower an individual’s intrinsic motivation to adopt healthy lifestyles, and has shown promise as a program that supports lifestyle changes [7–9]. More specifically, MI practitioners often utilize four core concepts (collaboration, evocation, autonomy, and empathy), as well as positive communication skills to support and facilitate a child’s self-efficacy for engaging in healthy behaviors [10, 11].

## MI Interventions to Promote Lifestyle Modifications

MI programs targeted at improving lifestyles are frequently implemented with adults with overweight/obese, but relatively few that focus on behavioral outcomes have been conducted with children. Among the few existing systematic reviews and meta-analyses on this topic [12–17], the primary interests examined the effects of MI on changes in body mass index (BMI) and/or other body fat distribution. Although existing qualitative synthesis suggests MI may improve healthy lifestyle behaviors [17], there are relatively few systematic reviews that have investigated the effects of MI on children’s lifestyle behavioral changes (eating habits and PA patterns). In fact, with limited reviews focusing on children’s lifestyle behaviors or included the behaviors as secondary outcomes, the pooled effects were not quantitatively assessed.

While multiple studies have verified the positive role of MI interventions regarding weight loss, their effects and those of potential moderators on children’s eating habits and PA remain unclear. Furthermore, understanding potential moderators of MI is critical as it can help develop more tailored MI interventions targeting children’s lifestyle behaviors [18]. According to Miller and Rollnick [19], MI practitioners’ training and background and the way in which MI interventions were delivered (in-person or remotely by telephone calls, texts, audio, or video) or implemented (as a core or an adjunct treatment), may modify the intervention effects [20, 21]. Potential moderators could include the duration of the MI program, number of in-person sessions, number of targeted lifestyle behaviors, format for delivery, whether it was a core or an add-on component to another existing program, and some important personal characteristics of participants, including children’s baseline weight and biological age [22]. Thus, in this systematic review and meta-analysis, the outcomes of interest were children’s obesogenic behaviors, including poor eating habits (e.g., ↓F/V, ↓dairy, ↑sugary beverages, ↑calorie, ↑snacks, and ↑fat intake) and poor PA patterns (e.g., ↓MVPA and ↑screen and ↑sedentary time).

## Evidence Acquisition

### Literature Search

**Fig. 1.**
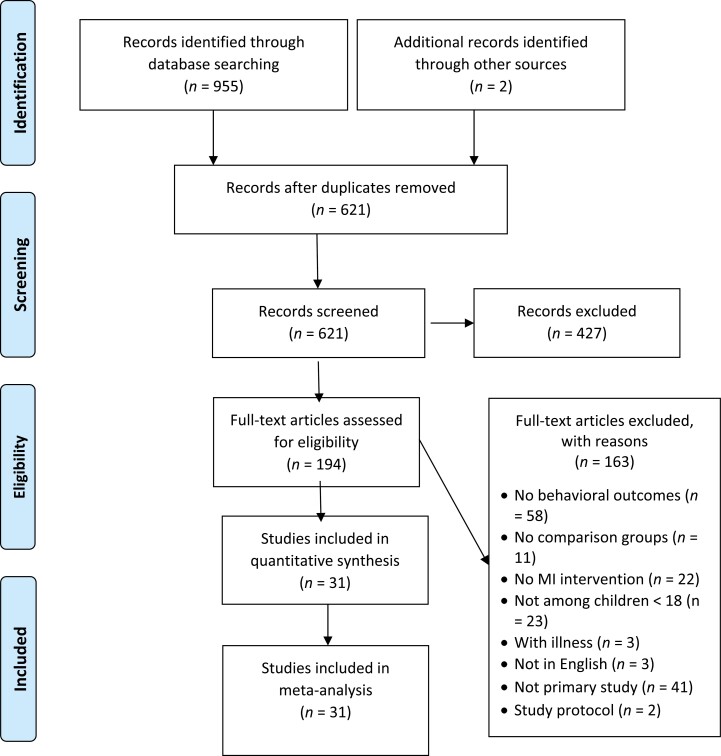
PRISMA flow diagram.

The Preferred Reporting Items for Systematic Reviews and Meta-Analyses (PRISMA) statement and checklist was followed for this review (see Fig. 1 and suppl checklist). With the assistance of our university’s health science librarian, one researcher (CV) conducted searches of the following six databases: CINAHL, Cochrane, PsycINFO, Embase, PubMed, and Web of Sciences; the search was confirmed by another researcher (RH) using the same search strategies. The following keywords and phrases were employed: (“motivational interview” OR “motivational interviewing”) AND (“physical activity” OR “physical activities” OR “physical fitness” OR “exercise” OR “weight loss” OR “diet” OR “Diet”[MeSH] OR “eat”) AND (“child” OR “pediatric” OR “parent” OR famil* OR adolescen*). We also used CINAHL and MeSH subject headings when appropriate. Articles were limited to English, with publication dates ranging from 2005 to 2022, as the application of MI in the prevention and treatment of childhood obesity emerged around 2006 [23, 24]. In addition, we reviewed the reference lists of published review articles for potential eligible articles. We then exported results to the Endnote X9 reference management software and removed duplicates before screening.

After removing duplicates (*N* = 316), 641 article abstracts were screened for potential inclusion. Studies were retained if they (a) utilized MI as a key or an important add-on component to the intervention program, (b) were designed to treat or prevent childhood obesity (programs targeting children’s BMI or body fat distributions), (c) involved a comparison/treatment group with two time points, (d) included child participants (aged ≤ 18 years), (e) reported at least one children’s lifestyle behavioral change (F/V, dairy, sugary beverages, calories, snacks, fat intake, MVPA, and screen time), and (f) reported enough information to calculate standard mean change in one of the targeted outcomes. To avoid potential confounders, articles were excluded if the study focused on adults only or on children diagnosed with posttraumatic stress disorder, attention deficit hyperactivity disorder, asthma, cancer, diabetes, thyroid dysfunctions, or eating disorders. We also excluded review articles and case studies. When multiple articles reported the same intervention, we retained the article with the largest sample size and reported outcomes relevant to this review.

## Data Screening and Extraction

This systematic review applied a two-step screening approach: (a) screening titles and abstracts and (b) screening full texts. The first author developed the inclusion/exclusion criteria for data extraction and evaluation. Guided by these criteria, two trained coders independently screened each article’s title and abstract for eligibility. The first author independently evaluated and verified all full-text articles deemed potentially eligible by at least one coder prior to extracting data from each retained article for the review. At each step of the evaluation, the two independent coders discussed their results with the first author to resolve any potential discrepancies until consensus was reached. Data on behavioral outcomes, comparison group, study design, sample size, mean age, MI as the core or add-on component, MI targets (child alone, parent alone, or parent-child dyads), number of sessions, duration of treatment, follow-up time, fidelity assessment, MI intervenor training, and key findings were extracted as presented in Table 1.

**Table 1:** Article Summary

Authors/years	Design/country	Child age *M* (*SD*); *N*	TX fidelity/MI training	Intervention focus	Outcomes	Key findings
1. Armstrong, 2018	2-group RCT; US Feasibility study Targeting parents	5–12 years 9.9 (9.2) *N* = 101	By a MINT trainer ***Tx fidelity checked***	Treatment program 12-week pre-post. 120 daily interactive text MI and 2–3 clinical visits. Clinic-Based	MVPA^a^ Screen time^a^ Veg^a^ Fruit^a^	- Interactive MI texts combined with clinical visits are feasible - Non-significant ↑MVPA (*p* = .72); ↓Screen time *(p* = .17) - Non-significant Δ in Veg and Fruit
2. Bean, 2018	2-group RCT; US Targeting adolescents	11–18 years 13.6 (1.8) *N* = 99	MINT; > 30 hr ***Tx fidelity checked***	Treatment program 3-month pre–post, and 6-month f/u. 2 MI sessions. Academic medical center	7-days MVPA^a^^,^^±^	-Significant ↑MVPA @ 3- and 6-month (*p* < .05) for both groups -Significant ↓energy intake @ 3-month, compared to control group
3. Black, 2010	2-group RCT; US Targeting Black adolescents	11–16 years 13.3 (1) *N* = 235	Mentors received 40-hr MI training	Prevention program Baseline (T1), 11-month (T2); 24-month (T3); 12 sessions over 10 months	PEPA (min/d)^±^ Energy (kcal)	-Significant ↑PAR (≥ 1800 count/min) per Actiwatch -T1 → T2, *F* = 17.09, *p* < .001; *η*_*p*_^2^ = 0.16 -T1 → T3, *F* = 12.89, *p* = .001; *η*_*p*_^2^ = 0.12
4. Broccoli, 2016	2-group RCT; Italy Sustainability study Targeting parent + OW child	4–7 years *N* = 372	20-hr MI training	Treatment program T1 (baseline), T2 (12-months) and T3 (24 months). 5 clinic visits. Clinic-Based	Non-organized PA^±^; eating habits^±^	-↑ non-organized PA Δ b/t groups (*p* = .07) @ T2, but not @ T3 -Sig improved eating habits (sweet, pop, fat, and dessert)
5. Chahal, 2014	2-group non-RCT; Canadacollaborative/didactic	10–16 years 11.1(3.5), *N* = 112, 51% female	Web-Based MI training. No details	Treatment program dyslipidemia management T1 (baseline), T2 (6-months); MI as an adjunct to a peer mentor program	MVPA^a^^,^^±^ (min/wk) Screen time^a^^,^^±^ Nutrition scores^a^^,^^±^	-Collaborative group > didactic group -Significant ↑ PA (+4.0 hr/week vs. +2.0 hr/week; *p* = .05) -Significant ↓screen time (−7.0 hr/week vs. +1.3 hr/week) -Significant ↑Nutrition scores (5.3/10 vs. 6.6/10, *p* = .004)
6. Chahal, 2017	2-group RCT; Canada Adolescent alone or with parent (dyad) family-centered	10–17 years 12.8 (2.2) 13.7(2.5) *N* = 32	MINT: 2–3 days training ***Tx fidelity checked***	Treatment program: dyslipidemia management T1 (baseline), T2 (6-month). 4 clinic visits (30–45 min) + 4 telephone calls (5–10 min). Clinic-Based	MVPA^b^ Veg^a^^,^^±^ Fruit^a^^,^^±^	- ↓MVPA^b^ in both groups -↑ veg and ↑ fruit in both groups -No significant difference in b/t group Δ
7. Cloutier, 2015	2-group non-RCT; US, parent-child dyad low-income mothers	2–4 years 2.98 (72) *N* = 418 82% Hispanic; 18% AA	by a MI trainer; > 2 hr ***Tx fidelity checked***	Treatment program T1 (Baseline); T2 (12 month), Clinic- Based	Sweetened-B^a^^,^^±^ Fruit^a^ andVeg^a^ Step^a^ and Screen^a^^,^^±^	Compared to control -Significant ↓milk and sweetened beverages (*p* < .05) -Significant ↓screen time (*p* < .05) -Significant ↑ juice (*p* = .001)
8. Currie, 2016	2-group RCT; US Targeting obese adolescents (≥ 95th %); PA only	12–18 years 14.4 (1.92) *N* = 64, 56% female	7-hr MI training ***Tx fidelity checked***	Treatment program 7-week MI (~50 minutes) telephone calls (9:05 ± 3:14 min)	Pedometer-accessed PA^b^ (steps)	-High attrition rate (63%) -Pedometer PA increased for both groups, no significant difference b/t groups (*p* >.05). -Significant ↓BMI for intervention group is related to other factors
9. Davis, 2011^76^	3-group RCT; US Targeting Latino adolescents	4–18 years 15.8 (1.1) *N* = 38	by 2 MINT trainers ***Tx fidelity checked***	Prevention program 16-week pre-post design with CT, MI+CT, and control groups. 32 CT sessions + 8 MI sessions. Community setting	ActiGraph^b^ (MVPA%)^±^ Energy (kcal/d) Fat Carbohydrate	- MI as an adjunct to circuit training (CT) - The MI+CT groups had sig ↑ MVPA and cardiovascular fitness compared to the control group
10. Davoli, 2013	2-group RCT; Italy Targeting parents	4–7 years 6.7(0.99) *N* = 374	20-hr MI training	Prevention program T1 (baseline), T2 (12-month); 5 family meetings. Clinic-Based: Pediatricians	PA^a^ habits^±^ Dietary habits^±^	- More *positive* lifestyle changes (parent-reported PA and dietary, in %) in the MI group. - Significant ↓ screen time, desserts, fried food, sweets, sweetened drink - Significant ↑ non-organized PA, vegetable soup
11. Dawson, 2014	2-group RCT; New Zealand Targeting parents	4–8 years 6.4 (1.4) *N* = 271	Online and 2-day MINT: 40 hr ***Tx fidelity checked***	Prevention program T1 (baseline), T2 (2 weeks). A single session of MI	PA^a^, Screen time Veg and sweetened drink	- No significant difference b/t groups in behavioral Δ - MI group has sig ↑ in autonomous motivation in lifestyle Δ
12. Doring, 2016	2-group RCT; Sweden Targeting parents	Assessed from 9–48 months of age 4.3(0.4) at f/u; *N* = 1,041	MI training mentioned, but no details	Prevention program T1 (baseline), T2 (39-month), 6 sessions + 1 group +2 calls. Clinic-Based	ActiGraph^b^ Veg^±^ Sweetened drink^±^	- No significant difference in children’s PA, MVPA (min/d) *p* = .81 - Small intervention effect in healthier dietary habits - Significant ↑veg and ↓ sweetened drink (*p* < .05)
13. Early, 2019	2-group quasi-experimental; US Targeting child or parent	2–18 years *M* = 10.8 *N* = 45	N/A	Prevention program T1 (baseline) T2 (3-month and T3 (6-month). 4 sessions. Home or clinical visits	PA^a^ Screen time Sweetened drink^±^	- Slight decrease in PA (*p* > .05) - Significant ↓ sweetened drink and ↑water (*p* < .05) - ↑veg, ↓screen time (but non-sig *p* > .05) - MI group outperformed control group
14. Gourlan, 2013	2-group RCT; France Targeting obese adolescents	11–18 years 13 (1.66) *N* = 54 (41% female)	32-hr MI training ***Tx fidelity checked*** using MITI code	Prevention program T1 (baseline) T2 (6-month) pre-post. standard weight loss program (SWLP) vs. SWLP + MI (6 telephone calls) groups. Referred by clinicians	Accelerometer-assessed PA^b^ Length^±^ Energy expenditure^±^	- MI as an adjunct to SWLP has increased autonomy and lower amotivation in PA (*d* = 0.44, *p* < .01) - MI+SWLP with sig ↑ in PA length (*d* = 0.39, *p* < .01), ↑ energy expenditure (*d* = 0.36, *p* < .01)
15. Kong, 2013	2-group RCT; US Targeting OW/OB adolescents	13–16 years 15 (1) *N* = 51	2-day MI workshop ***Tx fidelity checked***	Treatment program T1 (baseline) T2 (9 month). 8 ACTION (MI + education) vs. UC. School-Based health clinics	Accelerometer MVPA^b^^,^^±^ 3DPAR MVPA Screen time^±^ Sweetened drink Veg/Fruit	- Significant b/t group Δ with ↑ in device-assessed MVPA (*p* = .008), but not self-reported 3DPAR MVPA (*p* = .63) - Significant b/t group Δ with ↓ screen time on weekdays (*p* = .03), but not on weekends (*p* = .17) - No significant b/t group Δ in sweetened drink, veg, or fruit (*p* > .05)
16. MacDonell, 2012	2-group RCT; US Targeting African American (AA) adolescent-caregiver dyads	13–17 years 15.05 (1.4) *N* = 44	16-hr MI training and weekly supervising ***Tx fidelity checked***	Prevention program T1 (baseline) T2 (10 weeks). MI vs. nutrition counseling groups. 4 sessions. Clinical-based	PA^a^ MET Fast food^±^ Soft drink Veg Fruit	- MI group has improved eating behaviors (↓fast food, ↑veg, ↓Fruit) and ↑ motivation toward PA
17. Neumark-Sztainer, 2010	2-group RCT; US Targeting adolescent girls (75% minority background)	15.8 (1.2) years *N* = 356, 75% female	MI training mentioned, but no details	Prevention program 1 academic year, and 9-month f/u. PE + MI + parent outreach schools vs. UC schools, 5–7 sessions. School-Based	PA^a^, sedentary time, screen time	- MI group has significant between-group difference in ↓ sedentary time (*p* = .05) and 13.7% ↓ unhealth weight control behavior - No significant Δ in PA (30 min/block) or screen time
18. Norman, 2019	2-group cluster RCT; Sweden Targeting parents	6 years 6.3(0.3) *N* = 215	MI training mentioned, but no details. ***Tx fidelity checked***	Prevention program T1 (baseline) T2 (8-month), T3 (5-month f/u) T4 (4-year f/u).1–2 sessions. School-Based	Organized PA^a^, screen time Unhealthy diet	- No b/t effect on dietary intake, screen time or PA - Significant ↓ unhealth food and drinks (*p* < .05) @ T4 - ↑Organized PA @ T4 (*B* = 1.77*, p* = .16)
19. Nyberg, 2015	2-group cluster RCT; Sweden, School-Based Parent of preschooler	6.2(.3) *N* = 243	***Tx fidelity checked*** using MITI codes	Prevention program T1 (baseline), T2 (8 months), T3 (added 3 months f/u)	Accelerometer MVPA^b^ (> 500cpm) Sedentary time	- Significant ↑ weekend PA for girls - Significant ↓ sedentary time in MI group @ T3
20. Nyberg, 2016	2-group cluster RCT; Sweden Targeting parent and child	6 years 6.3(0.3) N = 268	***Tx fidelity checked*** using MITI codes	Prevention program T1 (baseline), T2 (6-month), and T3 (5-month f/u). 1–2 sessions. School-Based	Accelerometer MVPA^b^ (> 500cpm) Veg/fruit Unhealth food	- Significant ↓ sedentary time @ T3 (*p* < .01) - No significant ↓ MVPA @ T2 and T3 (*p* > .05) - Significant ↓ unhealth food, snacks, drink @ T2 (*p* < .05) - No significant dietary Δ @ T3
21. Ogu, 2014	2-group non-RCT; US Targeting Hispanic parents	2–4.5 years 3.1 (1.2) *n* = 15 3.2 (0.6) *n* = 15	N/A	Prevention program T1 (baseline), T2 (6-month). 4 visits. For WIC families Community setting	PA^a^ Veg/fruit Unhealth food	- Significant ↑ in Veg intake in MI group (*p* =.013) - MI group has better dietary patterns - No significant Δ in milk, sweetened drink, fruit intakes - No significant Δ in PA - Significant ↑ PA goals
22. Olson, 2008	2-group non-RCT; US	11–20 years, *n* = 284; 45% < 14 years	3-hr MI training ***Tx fidelity checked***	Prevention program T1 (baseline), T2 (6-month). Clinic-based	PA^a^ Milk product	- Significant ↑ PA (30 min/day) in MI group - Significant ↑ motivation/action for healthy lifestyles in MI group
23. Pakpour, 2015^27^	3-group RCT; Iran MI with obese adolescent alone or MI + Parent Involvement (PI), or UC	13–18 years 15.78(1.19) *N* = 357	***Tx fidelity checked*** using MITI codes	Prevention program T1 (baseline), T2 (12-month f/u). MI or MI+PI vs. UC, 6 sessions. Clinic setting	MVPA^a^^,^^b^^,^^±^ Dietary intake^±^	- Significant dietary and PA improvement in both MI+PI and MI alone (*p* < .01) in relation to UC - MI + PI group outperformed MI alone in fat, snacks/desserts, fried food (*p* < .05) - Significant ↑ MVPA (length and energy) in self-report and device-assessed
24. Pfeiffer, 2019	2-group cluster RCT; US; School-Based	12.05 (1.02) *N* = 1519	2-day MINT (16 hr) ***Tx fidelity checked*** using MITI codes	Prevention program T1 (baseline), T2 (17 week), and T3 (9-month f/u). 17 weeks intervention, 2 MIs	MVPA^b^ (> 2296 counts/min) Aerobic performance	- Aerobic performance ↓ in both groups but less with MI group - No significant Δ in MVPA
25. Resnicow, 2016 [62]	3-group cluster RCT; US Targeting parents	2–8 years *M* = 5.1 *N* = 467	1.5-day MI training ***Tx fidelity checked*** using MITI codes	Prevention program T1 (baseline), T2 (2-year); PCP only (3 + 1 booster), PCP(4) +RD (6 session) + SMS, vs. UC, Clinic-based.	PA^a^ (hrs/d) Screen time^a^ Veg/Fruits^a^	- PCP + RD group with ↑PA, ↑ fruit, ↑veg, ↑sweetened drink, ↑ eating out, ↑screen time, ↑snacks - PCP group with ↓PA, ↑ fruit, ↑veg, ↑sweetened drink, ↑ eating out, ↓screen time, ↑snacks
26. Robson, 2019	3-group RCT; US LAUCH (23 hr of weight loss txt), MI (7.5 hr), UC Targeting parents	Preschoolers 4.59 (0.93) *N* = 104	N/A	Treatment program T1 (baseline), T2 (6-month). Clinic- and home-based. MI: 4 in-person, 14 calls	24-hr dietary recall	- LAUCH outperformed MI and UC in “red” food (*p* < .05), but no difference in “green” food - Different effects may be due to different dosing (23 vs. 7.5 hr), not approach
27. Schwartz, 2007	3-group non-RCT, US, Targeting OB parents and OW children	3–7 years *M* = 5.3 *N* = 91	2-day MI training	Prevention program 6-month pre-post. UC, 1 MI from PCP, or 2 MIs from PCP + RD; 4–5 MIs Office-Based clinic	Food frequency Sweeten-drink Snacks/desserts^±^ Dining out^±^	- Significant ↓ snacks and desserts in Mini-MI group (*p* <.01) vs. control - Significant ↓ dinning out in intensive MI vs. Mini-MI group
28. Taveras, 2011	2-group cluster RCT with 10 pediatric practices, US Targeting children	2–7 years 4.9 (1.2), *N* = 475	***Tx fidelity checked***	Prevention program 1-year pre-post. 4 sessions + modules + 3 telephone calls. Clinical setting	PA^a^ Screen time^±^ Sweetened drink Fast food	- Significant ↓ in screen time (-0.36 hr/day, *p* =.01) vs. control - Significant ↓ in sweeten-drink and fast food (p >.05)
29. Tucker, 2013	2-group quasi-experimental Targeting parents and obese child US	4–18 years; *M* = 9.5 and *M* = 9.9; *n* = 96 Implementing Let’s Go 5-2-1 program with MI or standard care	3-day MI training ***Tx fidelity checked***	Prevention program 6-month pre-post and 12-month f/u. SC + 1 MI session. Clinical setting.	PA^a^; screen time; Fruit and Veg^±^; dairy	- Significant ↓ in screen time (*p* < .05) in MI group over time - Significant ↑ in water intake (*p* < .05) in MI group over time - Significant ↑ Veg and Fruit (*p* < .01) in MI group over time
30. Wong, 2013	3-group quasi-experimental Targeting obese children; Hong Kong	Grades 5–6 *N* = 185	RN received MI training, but no other details.	Prevention program 14-week pre-post. MI, MI + parent (telephone calls) vs. UC; 5–6 sessions. School-Based	PA^a^ Calorie	- Significant ↓ Diet intake (calorie) in MI and MI+ groups over time - Significant ↑ PA in MI and MI+ over time
31. Zanatta, 2020	2-group RCT; Overweight/obese adolescents; Brazil	15–18 years; 17.3 (1.0), 16.8 (0.9); *N* = 37	N/A	Prevention program 12 weekly sessions T1 (baseline), T2 (3 months)	PA^a^^,^^b^ (self-report and pedometer)	- No significant difference b/t groups in exercise capacity and PA in self-reported or pedometer assessed PA

*Tx fidelity* treatment fidelity; *b/t* between; *Sig* significant; *MINT* Motivational interviewing network of trainers; *MITI* Motivational Interviewing Treatment Integrity; *AA* African American.

^a^Self-reported.

^b^Device-assessed Δ = changes.

^±^With statistically significant outcome change (*p* ≤ .05).

## Data Analysis

The Comprehensive Meta-Analysis software was used to conduct the meta-analysis. Hedges *g*, rather than Cohen’s *d*, was calculated as the effect size using random effect models because Hedges *g* is more sensitive to small sample sizes [25, 26]. For interventions with a three-arm design, the findings of both MI arms (relevant to the control group) were included in the meta-analysis. The effect size for each behavioral outcome was calculated and verified by the first and second author using the mean, standard deviation (*SD*), and sample size at each time point (pretest, posttest, and follow-up). For articles that did not provide the needed statistical data, the corresponding authors were contacted by email twice (2 weeks apart) to obtain relevant information. For a few studies that reported medians and interquartile ranges (IQRs), corresponding authors were contacted twice to obtain means and *SD*s first. However, if a response was not received, recommended calculations for means [27] and *SD*s [28] were conducted using reported sample sizes, medians, and IQRs. The *Q* test and *I*^2^ statistics were performed to determine the degree of heterogeneity in the included studies [29, 30]. Moderation analyses were conducted with mixed-effects models to examine if study and intervention characteristics (e.g., child’s age, baseline BMIs, intervenor, intervention duration, study setting) moderated the intervention effects. To evaluate publication bias, the results from the Begg and Mazumdar rank correlation test, Egger’s regression asymmetry test, and funnel plot were used.

In addition, sensitivity analysis was performed to examine whether the effect sizes varied by studies’ risk of bias (low risk vs. moderate to high risk). The optimal information size (OIS) was estimated for each outcome using the random-effects power analysis method developed by Valentine and colleagues [31]. Each outcome’s pooled effect size, average number of participants in each group, total number of effect sizes, and level of heterogeneity among included studies were used to estimate the observed power in this meta-analysis.

## Risk of Bias Assessment

We used the Cochrane Collaboration’s tool [32] to assess the risk of bias for each included article. The tool includes six quality indicators: random sequence generation (one item), allocation concealment (two items), blinded outcome assessment (two items), clear explanation for dropouts (one item), incomplete data (two items), and selective report/other biases (three items). For each quality indicator, risk of bias was categorized as (0) *very low risk*, (1) *low risk*, (2) *moderate risk*, and (3) *high risk*; and we calculated a final average risk of bias score from the six quality indicators. Studies were evaluated to have moderate to high risk of bias if at least three quality indicators were scored as moderate risk or at least one quality indictor was scored as high risk. The first author and two evaluators independently rated each included study and met several times to resolve any potential disagreements on scoring. Supplementary Table 1 illustrates the quality appraisal ratings for all retained articles.

## Evidence Synthesis

### Description of Studies

Of the 194 full-text articles screened in the review, 163 were excluded because they did not meet the eligibility criteria (see Fig. 1). Among the eligible 31 studies, more than half were conducted in the USA (*s* = 17, 55%), followed by Europe (*s* = 8, 26%) [33–40], Canada (*s* = 2, 6%) [41, 42], Brazil (*s* = 1, 3%) [43], Hong Kong (*s* = 1, 3%) [44], and Iran (*s* = 1, 3%) [45]. The 31 studies included 19 randomized controlled trials (RCTs), four cluster RCTs [38–40, 46], and eight quasi-experimental designs [42, 44, 47–52]. The majority of studies (*s* = 22, 71%) were designed to prevent obesity, while nine studies focused on weight management [29, 30, 33, 41, 42, 53–56]. These 31 studies included a total of 8,499 children (range: 32–1,519 [37, 42] participants per study) with a mean child age of 8.838 years (*M*_age_ = 2.95–17.3 years). About 63% of the children (*N* = 5,152) were females. Only five studies reported parents’ age (*M* = 32.46 years). Participating children’s baseline mean BMI was reported as BMI-percentage = 82.95%, BMI *z*-score = 0.83, and BMI = 21.23 kg/m^2^. Fourteen studies targeted children alone, nine targeted parents, and eight targeted parent-child dyads as MI receiver.

Greater than three-quarters of studies (*s* = 24, 77%) targeted both behavioral outcomes (eating and PA), while fewer than one-sixth (13%) focused on one behavior only, either eating (*s* = 3) or PA (*s* = 4). Children’s PA was assessed by self-reported questionnaires from parents or children (*s* = 17) and/or activity devices worn by children (*s* = 12).

## Intervention Characteristics

Interventions were mostly conducted at clinics (*s* = 21, 67%) [33–37, 41, 42, 44, 45, 50, 52–54, 57], followed by schools (*s* = 6) [38–40, 46, 47, 58] and community settings (*s* = 4) [43, 54, 56, 60, 61]. Mean intervention length was 9.13 months (range: 0.5–39 months), with 10 studies conducting post-intervention follow-ups [38–40, 45, 46, 48, 53, 60, 63] for various behavioral outcomes. Intervenor background included trained primary care providers (PCP), registered dieticians (RD), registered nurses (RN), and student research assistants (RA). The MIs were mostly delivered by trained RAs (*s* = 12) [38–40, 43, 46, 49, 51, 53, 57, 60, 61, 63] followed by a PCP alone (*s* = 6) [33–35, 37, 50, 56], or a PCP plus RD or RN (*s* = 6) [45, 50, 52, 54, 55, 59] and an RN (*s* = 7) [36, 39, 41, 44, 47, 48, 58]. The MIs were delivered in-person (*s* = 17, 55%), or as a mix of in-person and booster sessions, such as telephone calls and online chats (*s* = 14, 45%). About 55% of studies (*s* = 17) used MI as the primary intervention component, while the remaining 45% (*s* = 14) incorporated other components to prevent or treat obesity. The majority of intervention periods were longer than 13 weeks (*s* = 24, 77%; *M* = 41), but had fewer than six sessions (*s* = 24, 77%; *M* = 4.097). Treatment fidelity evaluations were conducted and described in 19 studies (61%) to various degrees. Among the 19 studies, six (32%) used Motivational Interviewing Treatment Integrity (MITI) codes to evaluate the quality of MI implementation. Among the 26 studies describing intervenors’ MI training, 17 (65.4%) reported receiving 2–40 hrs. of MI training, while nine studies did not provide any training details (see Table 1). Reported types of MI training included the Motivational Interviewing Network of Trainers (MINT) training (*s* = 6), workshops (*s* = 4), online (*s* = 1) training, and certification (*s* = 1).

## Risk of Bias Assessment

Of the 31 included studies, no study was identified as having a high risk of bias (*M* = 2.01–3.0/3.0), whereas 24 (77%) had a low to moderate risk (*M* = 1.0–2.0/3.0). The remaining seven (23%) had a very low risk of bias (*M* = 0.67–0.83/3.0) [42, 47, 50, 55, 57, 58, 63]. The majority of the studies had a low-moderate risk of bias in six domains. The moderate risks of bias were mostly due to selection bias (random sequence or allocation concealment), performance bias (blind outcome assessment), and attrition bias (incomplete data). Since this systematic review was not limited to RCTs only, the selection bias is somewhat anticipated. No study was excluded as a result of risk of bias (see Supplementary Table 1).

## Publication Bias

As shown in Supplementary Figs., the funnel plots were all symmetric. No obvious publication biases were observed for consumption of F/V (Tau = −0.31, *p* = .143; *b* = −2.35, *t* = 1.74, *p* = .110), dairy (Tau = −0.24, *p* = .325; *b* = −2.33, *t*=1.58, *p*=.153), calories (Tau = −0.14, *p* = .652; *b* = −0.52, *t* = 0.27, *p* = .801), sugary beverages (Tau = −0.11, *p* = .469; *b* = −0.09, *t* = 0.06, *p* = .951), snacks (Tau = −0.20, *p* = .421; *b* = −1.42, *t* = 1.06, *p* = .322), fast food (Tau = 0, *p* = 1.00; *b* = 14.02, *t* = 1.22, *p* = .347), and fat (Tau = −0.07, *p* = .851; *b* = −3.61, *t* = 0.22, *p* = .838). In addition, there was no strong evidence for publication biases for MVPA (Tau = 0.24, *p* = .102; *b* = 1.26, *t* = 1.76, *p* = .092), energy expenditure (Tau = 0.33, *p* = .497; *b* = 18.28, *t* = 0.77, *p* = .523), and screen time (Tau = −0.31, *p* = .096; *b* = −1.54, *t* = 1.59, *p* = .134).

## Immediate Intervention Effects

The overall effects on ↑F/V (*k* = 13) were 0.10 (*p* = .334), and the heterogeneity was large with an *I*^2^ of 78.37% (*Q* = 55.49, *p* < .001). Moreover, the pooled effects were 0.10 (*p* = .056) for increasing vegetable intake (*k* = 11) and 0.02 (*p* = .663) for increasing fruit intake (*k* = 10). For the dairy intake (*k* = 11), one influential outlier [51] was identified (residual = 3.71, *∆I*^2^ = 34.08%) and removed. The effects on ↑dairy intake decreased from 0.08 (*p* = .426) to 0.02 (*p* = .724).

**Fig. 2.**
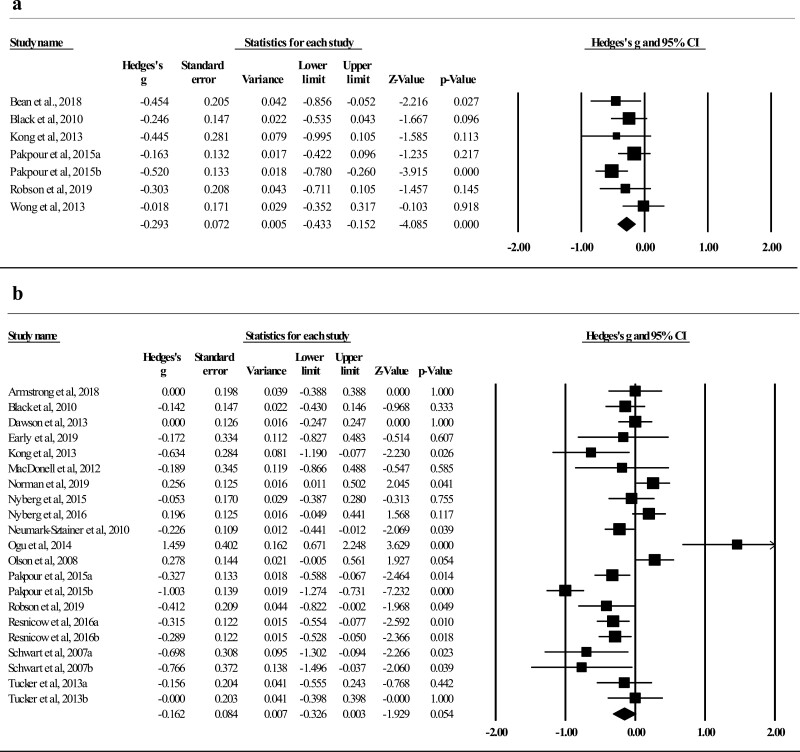
(a) Effects on decreasing calorie intake (*k* = 7). (b) Effects on decreasing sugary beverages (*k* = 21).

**Fig. 3.**
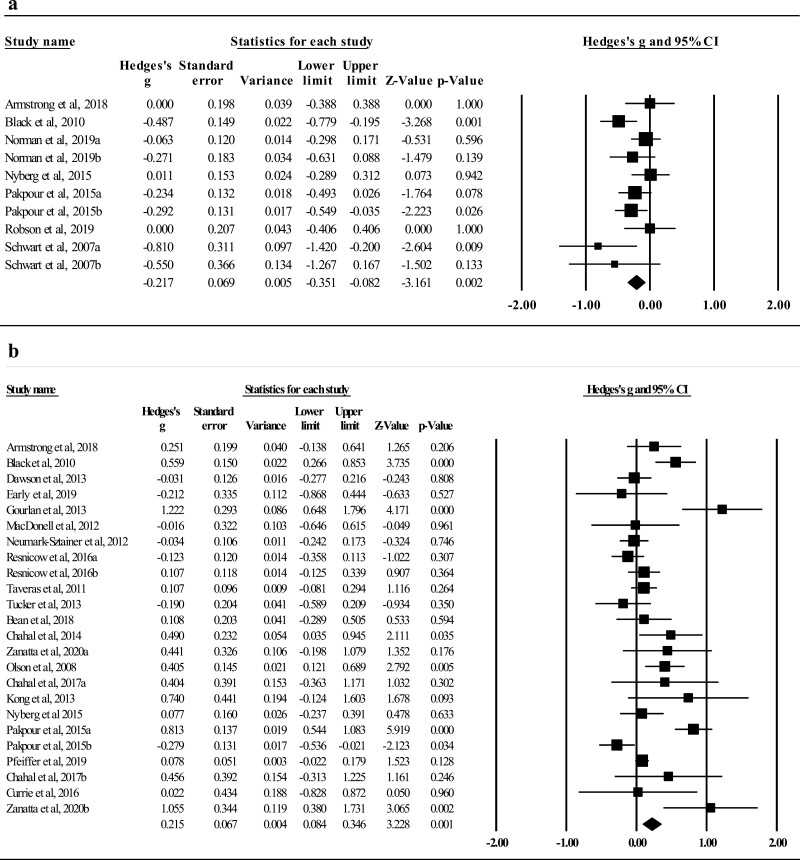
(a) Effects on reducing snacks intake (*k* = 10). (b) Effects on increasing MVPA (*k* = 24).

The pooled effects on reducing caloric intake (*k* = 7) were −0.29 (*p* < .001; see Fig. 2a), with small heterogeneity among the seven comparisons (*Q* = 7.50, *p* = .277; *I*^2^ = 20.02%). For decreasing consumption of sugary beverages (*k* = 22), one influential outlier [58] was removed (residual = −3.31, *∆I*^2^ = 15.52%). The overall effects were −0.16 (*p* = .054; see Fig. 2b) with strong heterogeneity (*Q* = 99.45, *p* < .001; *I*^2^ = 79.89%). The overall intervention effects on reducing snack intake (*k* = 10) were −0.22 (*p* = .002, see Fig. 3a), with small heterogeneity among the 10 comparisons (*Q* = 14.31, *p* = .112; *I*^2^ = 37.10%). Four studies, with high heterogeneity (*Q* = 226.88, *p* < .001; *I*^2^ = 98.68%), assessed fast food intake with a pooled effect of −1.20 (*p* = .245). The pooled effects on reducing fat intake were −0.20 (*p* = .044) with medium heterogeneity (*Q* = 16.04, *p* = .007; *I*^2^ = 68.82%; Supplementary Fig. 3).

For increasing MVPA (*k* = 24), the intervention effects were 0.22 (*p* = .001; see Fig. 3b), and the heterogeneity was medium with an *I*^2^ of 73.75% (*Q* = 87.60, *p* < .001). The pooled effects on ↑energy expenditure (*k* = 4) were 2.23 (*p* = .032) with strong heterogeneity (*Q* = 274.98, *p* < .001; *I*^2^ = 98.90%). In addition, the overall effects on ↓screen time (*k* = 16) were −0.06 (*p* = .176; Supplementary Fig. 4).

## Moderation Effects

Exploratory moderation analyses were conducted for the outcomes of F/V (*k* = 13), sugary beverages (*k* = 22), snacks (*k* = 10), dairy (*k* = 11), MVPA (*k* = 24), and screen time (*k* = 16). Interventions without a booster session had greater effects on ↑F/V (0.37 vs. −0.06, *p* = .026) and ↓snacks (−0.28 vs. 0.01, *p* = .023) than those with a booster session (Supplementary Tables 2 and 3). However, for ↑dairy intake, interventions with a booster session resulted in a greater effect than those without a booster session (0.17 vs. −0.08, *p* = .023; Supplementary Table 4). Moreover, interventions with a fidelity check resulted in greater effects in ↑dairy intake than those without a fidelity check (0.29 vs. −0.15, *p* = .014). As shown in Supplementary Table 5, when the number of MI sessions increased by one session, the intervention effects on sugary beverages decreased by 0.06 (*p* = .027). Similarly, the effects on ↓snacks were reduced when the number of MI sessions increased by one session (*B* = −0.04, *p* = .010). In addition, clinic-based interventions resulted in greater effects in ↑dairy intake than non-clinic-based interventions (0.12 vs. −0.14, *p* = .027). MI as an add-on to an existing program had better effects on ↑dairy intake than those that did not provide MI as an add-on (0.09 vs. −0.21, *p* = .034).

For ↑MVPA, interventions had better effects for children ≥ 11 years old (0.35 vs. 0.01, *p* = .015) and for those with obese (0.34 vs. 0.08, *p* = .039). As expected, intervention effects on ↓screen time increased with longer intervention duration (*B* = −0.007, *p* = .002; Supplementary Tables 6 and 7). In addition, MI interventions focusing on obesity treatment resulted in better effects on ↓snacks, ↑dairy, ↓sugary beverages, ↑MVPA, and ↓screen time than those targeting obesity prevention; however, these results were not statistically significant.

## Long-Term Follow-Up Effects

Only a few studies included a follow-up assessment. Two studies assessed F/V intake with an overall effect of −0.18 (95% CI [−0.42, 0.06]; *p* = .143). Similarly, two studies measured vegetable intake with an effect of −0.17 (95% CI [−0.38, 0.03]; *p* = .096). The pooled follow-up effects on increasing dairy intake were −0.13 (95% CI [−0.43, 0.17]; *p* = .399, *k* = 4) and −0.10 on decreasing calorie intake (95% CI [−0.40, 0.20]; *p* = .508, *k* = 2). For consumption of sugary beverages and snacks, the effects were 0.03 (95% CI [−0.17, 0.23]; *p* = .767, *k* = 5) and −0.01 (95% CI [−0.17, 0.16]; *p* = .956, *k* = 4), respectively. Moreover, two studies assessed fat intake with a pooled effect of 0.02 (95% CI [−0.19, 0.22]; *p* = .878).

Six studies assessed the follow-up effects on increasing MVPA; the overall effects were −0.04 (95% CI [−0.20, 0.12]; *p* = .611). For decreasing screen time, the follow-up effects were 0.12 (95% CI [−0.08, 0.33]; *p* = .242, *k* = 4).

## Sensitivity and Power Analysis

Overall, pooled effect sizes did not vary significantly according to studies’ risk of bias. Although not statistically significant, studies with moderate to high risk tended to have a greater effect size on increasing dairy intake than those with low risk of bias (0.17 vs. −0.06, *p* = .051). However, effect sizes on ↑F/V (0.02 vs. 0.14, *p* = .611) or ↓sugary beverage intake (−0.08 vs. −0.21, *p* = .426) were smaller among studies with moderate to high risk.

The average number of participants per group ranged from 77 to 128 among the outcomes. This meta-analysis had adequate power of estimating the effect sizes for calorie (98.61%), snacks (96.91%), and MVPA (99.99%). Although the total number of effect sizes for fast food and energy expenditure was only four, the observed power was about 100% due to the large, pooled effect sizes (*g* = −1.20 and 2.23). The observed power for F/V, dairy, sugary beverages, fat, and screen time was 24.23%, 5.5%, 68.98%, 75.97%, and 38.38%, respectively. To obtain at least 80% power, the total number of effect sizes or studies need to be 65 for F/V, 600 for dairy, 27 for sugary beverages, seven for fat, and 45 for screen time.

## Discussion

Obesogenic behaviors (↓F/V, ↓dairy, ↑sugary beverages, ↑fat, ↑screen time, and ↓PA) are identified as obesity risks [64]. Childhood obesity is a serious problem that puts children and adolescents at increased risk for chronic diseases and poor health outcomes. This review summarized the range of MI interventions designed for preventing/treating childhood obesity and examined their effectiveness in reducing obesogenic behaviors (↓F/V, ↓dairy, ↑sugary beverages, ↑fat and sedentary PA) [65]. Results from this systematic review and meta-analysis suggest that MI interventions produce small effect sizes (0.10–0.29) across a range of healthy eating patterns (↑F/V, ↓sugary beverages, ↓calorie, ↓snacks, and ↓fat) and PA (↑MVPA), and large effects (1.20–2.23) on ↓fast food and ↑energy expenditure. This indicates that MI outperforms the alternative (usual care) for a period (3–6 months) immediately after the interventions. The small effect on ↓sugary beverages noted in this meta-analysis (−0.16) is relatively lower to a recent meta-analysis focusing on adolescents weight management process (SMD = −0.47, *k* = 3, *I*^2^ = 26.2%) [66]. The effect of MI on children’s MVPA (0.22, *p* < .05) is significantly better than a recent meta-analysis assessing the effect of MI delivered in primary care settings with adults during a period of 6 months (0.04, 95% CI = −0.06 to 0.14) [67].

Although most effect sizes were small, statistically significant changes on ↓calorie, ↓snacks, ↓sugary beverages, ↓fat, ↑MVPA, and ↑energy expenditure were noted (*p* < .05). However, the changes on children’s ↑F/V, ↑dairy, ↓fast food, and ↓screen time are not statistically significant (*p* > .05). These insignificant findings can be related to the small number of studies, lower power, and high levels of heterogeneity among the studies. They may also be due to children’s (or proxy parents’) lower prioritization of behavioral modifications (less important dietary changes). It is also possible that, for weight management, health care providers may have consulted children to start with the reductions in calorie, snacks, and sugary beverages, while at the same time improving MVPA and energy expenditure for a quick weight reduction. It is possible that with a longer-duration program, providers could further consult children with greater intakes of healthy foods (↑F/V, ↑dairy, ↓fast-food) and minimize sedentary lifestyles (↓screen time). The above findings endorse the significance of continuously using MI techniques in the clinical setting to change children’s lifestyle behaviors.

From this meta-analysis, it appears that the intervention effects on children’s behavioral changes only lasted for a short period of time. This may be due to children’s minimal control over their food choices and/or PA, even if their motivation toward lifestyle changes increased. The behavioral changes at familial, parental, and societal levels may need to happen as well. For example, *familial obesogenic environment*, which is defined as a family environment promoting high-energy intake or a sedentary lifestyle, has been noted to significantly contribute to children’s poor eating patterns, sedentary lifestyle, and subsequently weight status [68]. Without adequate familial, parental, and societal support, children’s motivation alone may not be sufficient to sustain positive behavioral changes over time.According to Self-Determination Theory, it is critical for children to develop autonomous motivation for healthy lifestyles in order to sustain behavioral changes [58]. To better sustain healthy lifestyles, future MI interventions should incorporate family strategies (e.g., family goal setting/attainment and collective-efficacy building) to help children and their parents develop and maintain autonomous motivation in the home setting [69, 70]. However, only a few interventions in this review aimed to modify children’s obesogenic environment via interventions with parents. Incorporating MI techniques to help parents cultivate a healthy family environment (family-centered or community-centered approaches) [71] may be helpful in facilitating and sustaining children’s healthy lifestyles.

Since limited studies (ranging from two to six) conducted long-term follow-up assessments, it is difficult to draw any conclusions about the long-term effects of MI on behavioral changes. However, with the discouraging effects observed from these few studies, additional efforts are needed to improve and sustain long-term effects. Future obesity prevention/treatment programs may need to investigate possibilities of combining MI with other approaches (e.g., mindful eating, yoga, family-centered and community-based programs) [72, 73] to assist children to establish lifelong healthy behavior lifestyle.

An important additional element for sustaining children’s healthy behaviors may include fostering children’s self-regulation in healthy eating and PA, as self-regulation has been found to be related to behavioral change maintenance [74]. Having adequate motivation to initiate lifestyle change may not be sufficient for children to sustain behavioral changes over time. In fact, research has shown that children must have the capacity for successful self-regulation, which comprises a series of important competencies; this includes their ability to control inner states or responses toward thoughts, attention, emotion, or performance [75]. Many of these competencies must be nurtured within the context of a supportive environment. For example, in a study with four European countries (*N* = 2,764, *M*_age_ = 13.2), adolescents’ self-regulation significantly mediated the relationship between positive family meal culture and healthy eating behaviors [76]. Thus, to improve program’s sustainability, future MI interventions should aim to incorporate and promote the development of these important competencies within the family context by enhancing children’s ability to set and attain realistic personal and family goals and, ultimately, achieve behavioral changes over time [77]. In addition, MI techniques are often used to strengthen personal efficacy skills, such as children’s self-efficacy in leading healthy lifestyles [78]. However, to further utilize MI techniques in childhood obesity prevention, future MI programs should broaden from an intrapersonal focus and incorporate ecological influences on children’s lifestyle behaviors [79]. For example, building children’s collective-efficacy in healthier lifestyles at school and community levels may be helpful in creating a healthier environment and subsequently preventing childhood obesity [80].

This review revealed some important moderators for intervention effects on children’s lifestyle behaviors, including the existence of boosters, fidelity evaluation, number of MI sessions, children’s age, and baseline weight. Compared to programs that did not employ boosters, program with boosters had greater effects on ↑dairy intake. But this was not the case for ↑F/V and ↓snacks because non-booster MIs outperformed those with booster sessions. This may be due to the incidence of booster sessions with significantly fewer in-person sessions examined in this review; some critical content (↑F/V and ↓snacks) may not be delivered with limited in-person sessions (insufficient dosing). Thus, to overcome this problem, future MI programs should consider emphasizing these important contents (↑F/V and ↓snacks) in the booster sessions. Future studies could investigate the effects of utilizing remote contacts (e.g., video, interactive texts, telephone calls) to strengthen program content and dosing. Also, additional investigation must differentiate the impact of MI via different delivery methods (direct or remote contacts).

In addition to these mixed findings, it appears that children who were older, particularly for those with ↑BMI at baseline, responded better to the MI interventions than those who were younger and with ↓BMI. This is unsurprising, as older children and those with higher baseline BMIs may have greater motivation and readiness (developmentally and psychologically) to make behavioral changes to reduce their current weight. These participant characteristics may explain children’s self-regulation-related problems, particularly those associated with long-term behavioral sustainability. It is possible that children with ↑BMI at baseline may have poor capabilities or competencies to harness cognitive, emotional, and motivational resources to achieve and sustain long-term lifestyle behavioral goals [81]. Timely and effective MI interventions may be the resources they need to improve their competencies.

Additional efforts, including environmental support, may be needed to improve these children’s capacity for successful self-regulation. Furthermore, programs with more MI sessions, those with MI as an additional component, those provided in a clinical setting, and those that had fidelity appraisals seemed to outperform others. Programs with fewer sessions that provided MI as a single component, those that were provided in a non-clinical setting, and those without fidelity assessments with regard to behavioral changes (↑dairy, ↓sugary beverage, ↑MVPA, and ↓screen time) were not as successful. Therefore, clinic-based, multiple-component MI interventions with fidelity assessment and an increased intervention dose are recommended for future research. Additional efforts to identify the optimal, adequate intervention dose and the role of MI as an adjunct component to another obesity prevention program are needed.

## Limitations

This systematic review and meta-analysis have several limitations. One limitation is a lack of consistent and objective measurement of lifestyle behavioral outcomes. In this review, children’s eating patterns included children’s F/V, dairy, sugary beverages, calorie, snacks, and fat intake. Children’s dietary patterns were mostly assessed by self-report or parental proxy report dietary instruments. These approaches are prone to inaccuracies because of recall or observation bias [82]. The lack of an objective (or standardized) measurement to assess children’s eating patterns resulted in high levels of heterogeneity, leading to lower power in assessing pooled effect size. Currently, there are very few objective instruments to evaluate children’s eating patterns. This is not as problematic for PA assessment, as there are validated objective instruments (i.e., accelerometer) that can be used to assess children’s PA. However, among 30 studies investigating PA changes in this review, only 40% (*s* = 12) utilized a device-assessed PA report, and the majority (*s* = 18, 60%) still employed self-report methods for PA assessment. The continued use of self-report for PA may be due to its relatively straightforward implementation and lower cost. Another limitation is that we only explored potential moderators on some outcome variables (F/V, dairy, snacks, sugary beverages, MVPA, screen time) that had data on at least 10 comparisons (*k* = 10–24; Supplementary Tables). Thus, the potential moderators for MI effects on children’s lifestyle behaviors, particularly ↓fast food, ↓calorie, and ↓fat, may remain unclear due to this underpowered exploration. More efforts are needed to understand potential moderators of MI effects on children’s lifestyle behaviors.

## Conclusions

Despite the limitations mentioned above, this is the first meta-analysis to explore the pooled intervention effects and potential moderators of MI effects on children’s behavioral outcomes within the context of pediatric obesity prevention and treatment to our knowledge. The results indicate that MI interventions targeting both PA and healthy eating behaviors (and also delivered at clinical settings), may have promise in improving lifestyle behaviors among older children and for children with ↑BMI at baseline. However, the intervention effects appear to be short-lived. More efforts are needed to generate long-term effects of MIs, particularly on healthy lifestyle behaviors that can decrease children’s risk of obesity-related comorbidities. Finally, although there is an increasing trend of evaluating treatment fidelity, only slightly more than half of the included studies reported fidelity evaluation. Further emphasis is needed to enhance programs’ replicability. As an increased number of studies are conducted in this field, more detailed analyses should be undertaken to provide a more nuanced understanding of MI’s effectiveness in producing children’s improved behavioral outcomes.

## Supplementary Material

kaad006_suppl_Supplementary_Material
