# Supplementary material for: Motivational Interviewing in Pediatric Obesity: A Meta-analysis of the Effects on Behavioral Outcomes
Source: Ann Behav Med. 2023 May 17;57(8):605–19. doi: 10.1093/abm/kaad006 (PMC10354860; doi:10.1093/abm/kaad006)
Supplement: kaad006_suppl_Supplementary_Material [file kaad006_suppl_supplementary_material.docx]

**Supplemental Figure 1.** Effects on increasing the intake of fruits and vegetables (*k* = 13)

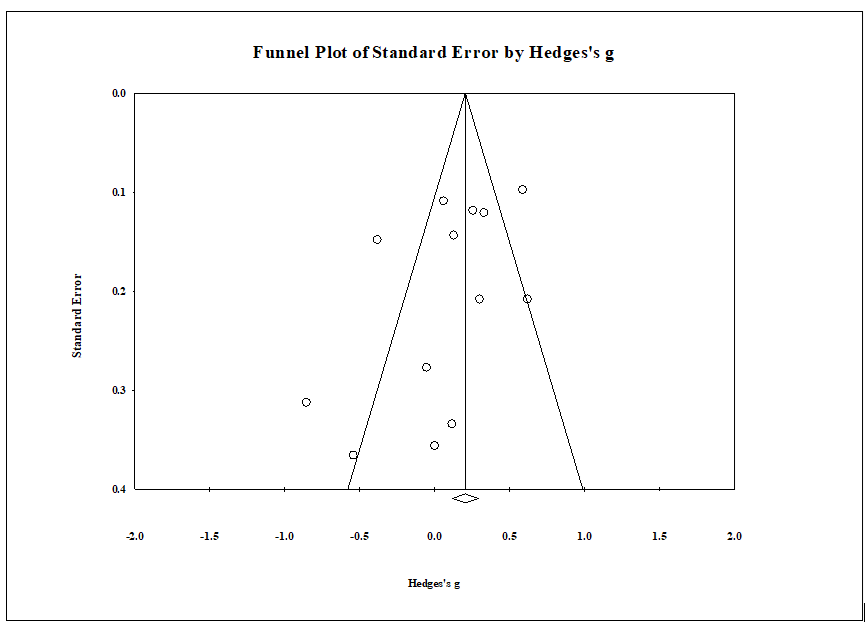


**Supplemental Figure 2.** Effects on increasing dairy intake (*k* = 10)

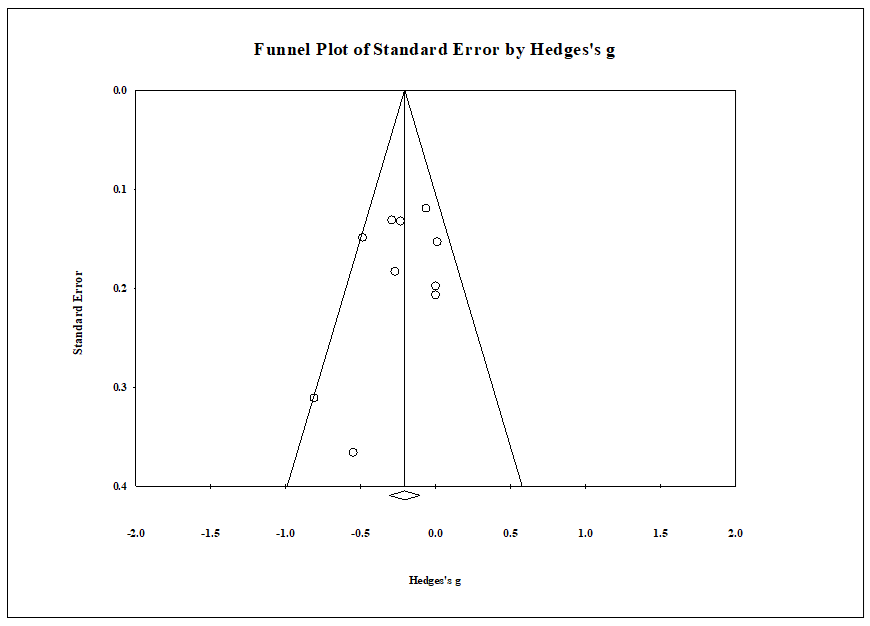


**Supplemental Figure 3.** Effects on reducing fat intake (*k* = 6)

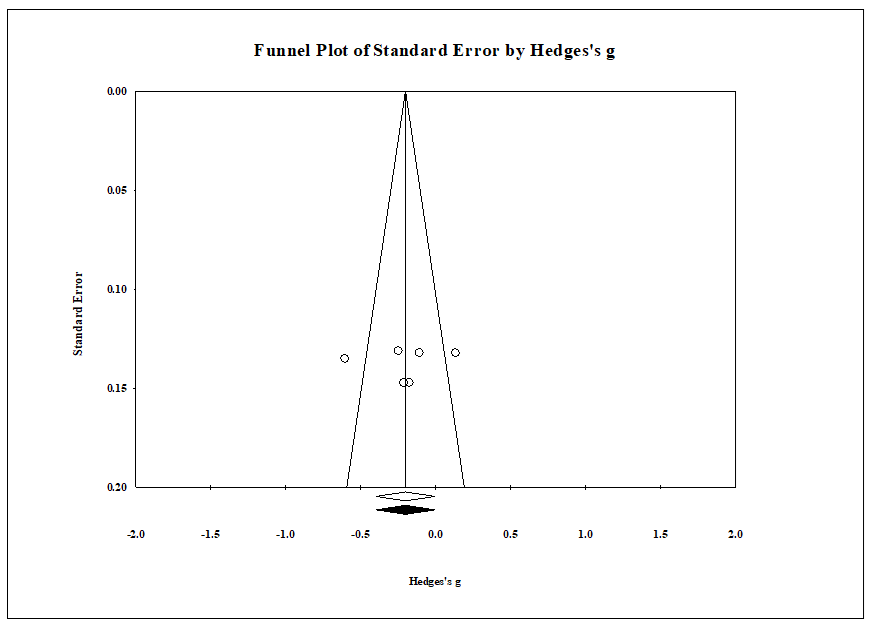


**Supplemental Figure 4.** Effects on decreasing screen time (*k* = 16)

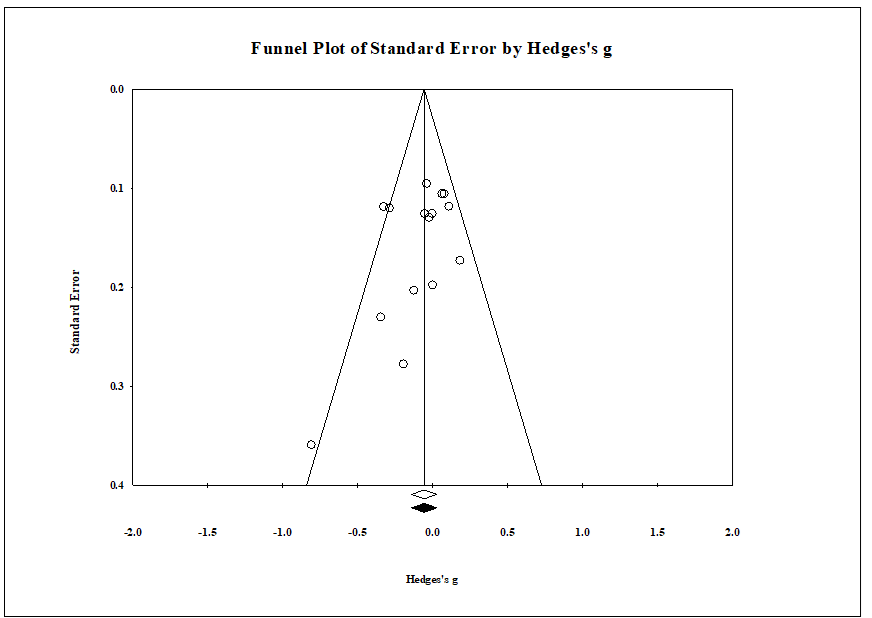


**Supplemental Figure *5:* Funnel Plots of Standard Errors for ↓Calories, ↓Sugary Beverages, ↓Snacks, and ↑MVPA**


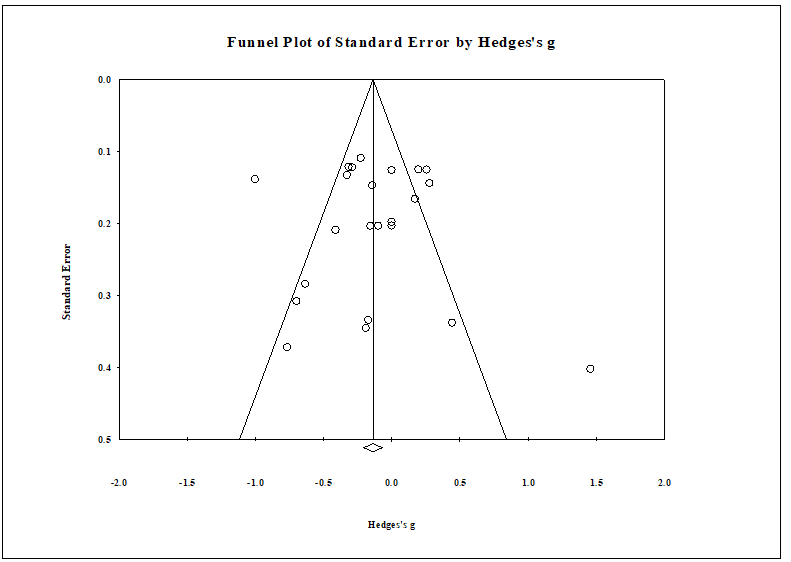

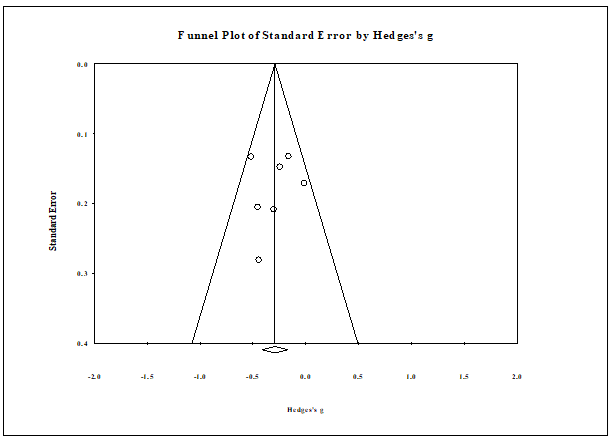


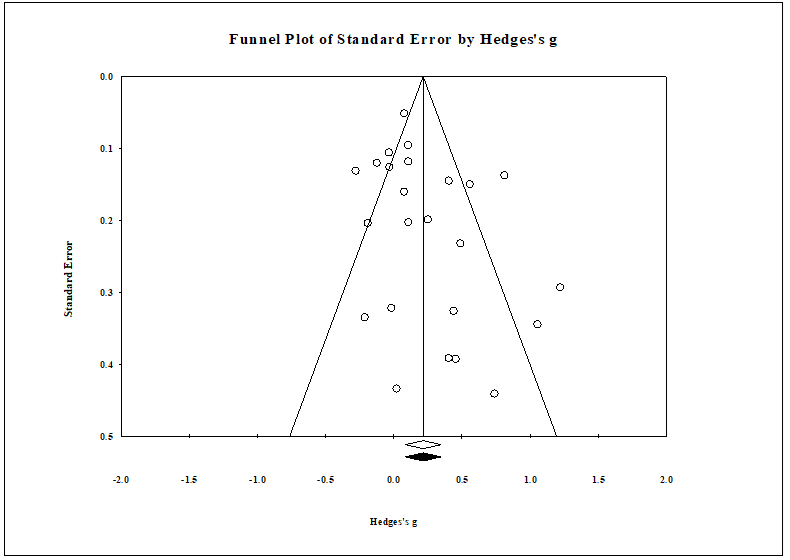

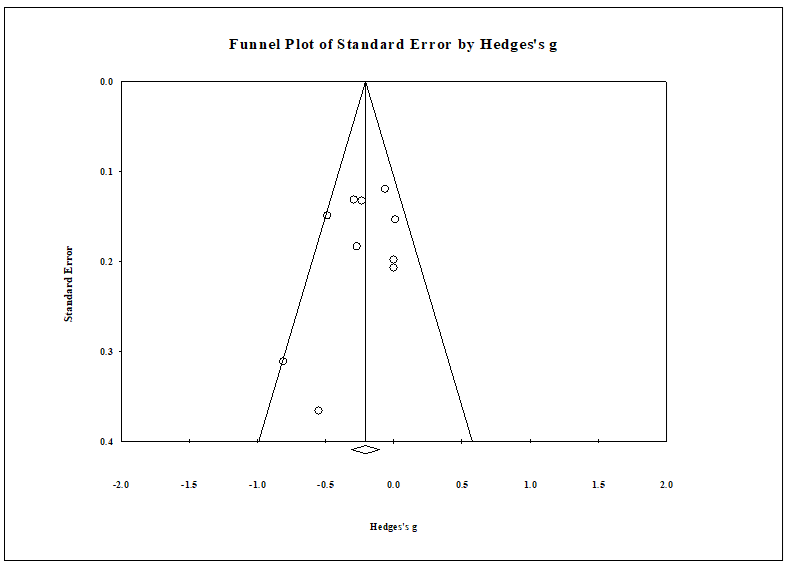


**↑MVPA**

**↓Sugary Beverages**

**↓Calories**

**↓Snacks**

**Supplemental Table 1**

Risk of Bias Assessment

| **Studies** | **Random sequence**  **(selection bias)** | **Allocation concealment**  **(selection bias)** | **Blinding outcome assessment**  **(performance bias)** | **Clear explanation of dropouts**  **(detection bias)** | **Incomplete data**  **(attrition bias)** | **Selective report**  **(reporting bias)** | **Overall Risk of Bias** |
| --- | --- | --- | --- | --- | --- | --- | --- |
| 1. *Armstrong, 2018* | 2 | 1 | 2 | 1 | 2 | 1 | Moderate |
| 1. *Bean, 2018* | 1 | 0 | 1 | 0 | 1 | 2 | Low |
| 1. *Black, 2010* | 1 | 1 | 2 | 0 | 0 | 2 | Low |
| 1. *Broccoli, 2016* | 1 | 2 | 2 | 1 | 1 | 1 | Low |
| 1. *Chahal, 2014* | 2 | 0 | 2 | 0 | 0 | 1 | Low |
| 1. *Chahal, 2017* | 1 | 1 | 1 | 1 | 1 | 1 | Low |
| 1. *Christie, 2017* | 1 | 1 | 1 | 1 | 1 | 1 | Low |
| 1. *Cloutier, 2015* | 1 | 1 | 2 | 0 | 0 | 1 | Low |
| 1. *Currie, 2016* | 1 | 1 | 1 | 1 | 3 | 2 | Moderate |
| 1. *Davis, 2011* | 2 | 1 | 2 | 1 | 2 | 1 | Moderate |
| 1. *Davoli, 2013* | 1 | 1 | 3 | 1 | 1 | 1 | Low |
| 1. *Dawson, 2013* | 1 | 1 | 1 | 1 | 1 | 2 | Low |
| 1. *Doring, 2016* | 1 | 1 | 2 | 1 | 1 | 1 | Low |
| 1. *Early, 2019* | 1 | 0 | 1 | 0 | 0 | 2 | Low |
| 1. *Gourlan, 2013* | 1 | 1 | 2 | 1 | 1 | 1 | Low |
| 1. *Kong, 2013* | 1 | 0 | 2 | 1 | 1 | 2 | Low |
| 1. *Macdonell, 2012* | 1 | 2 | 1 | 0 | 0 | 1 | Low |
| 1. *Neumark-Sztainer, 2010* | 1 | 2 | 2 | 1 | 1 | 1 | Low |
| 1. *Norman, 2019* | 1 | 0 | 2 | 1 | 1 | 2 | Low |
| 1. *Nyberg, 2015* | 2 | 1 | 2 | 1 | 1 | 2 | Moderate |
| 1. *Nyberg, 2016* | 2 | 1 | 2 | 1 | 1 | 2 | Moderate |
| 1. *Ogu, 2014* | 2 | 0 | 2 | 0 | 0 | 2 | Low 1 |
| 1. *Olson, 2008* | 2 | 2 | 2 | 2 | 2 | 2 | High |
| 1. *Pakpour, 2015* | 2 | 0 | 1 | 2 | 1 | 1 | Low |
| 1. *Pfeiffer, 2019* | 1 | 1 | 2 | 0 | 1 | 1 | Low |
| 1. *Resnicow, 2016* | 1 | 1 | 2 | 1 | 2 | 1 | Low |
| 1. *Robson, 2019* | 1 | 1 | 2 | 0 | 0 | 1 | Low |
| 1. *Schwartz, 2007* | 1 | 2 | 2 | 2 | 0 | 2 | Moderate |
| 1. *Taveras, 2011* | 1 | 1 | 2 | 0 | 0 | 1 | Low |
| 1. *Tucker, 2013* | 2 | 2 | 2 | 1 | 2 | 1 | Moderate |
| 1. *Wong, 2013* | 1 | 2 | 2 | 0 | 1 | 1 | Low |
| 1. *Zanatta, 2020* | 2 | 1 | 2 | 1 | 2 | 2 | Moderate |

*Note.* 0= No Risk; 1=Low Risk; 2=Moderate Risk, 3=High Risk

**Supplemental Table 2**

*Moderators for the Effects of Fruit and Vegetable Intake (k = 13)*

| **Continuous moderators** | **k** | **Slope B** | **SE** | **Tau^2^** | **Q_between_** | **p-value** |
| --- | --- | --- | --- | --- | --- | --- |
| Intervention duration | 13 | .006 | .006 | .09 | .98 | .323 |
| MI sessions | 13 | -.05 | .03 | .07 | 2.69 | .101 |
| **Categorical moderators** | **k** | **Effect size** | **95% CI** | **Q_between_** | **p-value** |  |
| Age |  |  |  | 2.10 | .147 |  |
| < 10 years | 8 | .22 | -.01, .45 |  |  |  |
| ≥ 11 years | 5 | -.05 | -.36, .72 |  |  |  |
| Baseline weight |  |  |  | .02 | .890 |  |
| Non obese | 8 | .08 | -.19, .36 |  |  |  |
| Obese | 5 | .12 | -.24, .47 |  |  |  |
| Purpose |  |  |  | .001 | .977 |  |
| Prevention | 10 | .10 | -.14, .33 |  |  |  |
| Treatment | 3 | .11 | -.38, .58 |  |  |  |
| Setting |  |  |  | 2.02 | .156 |  |
| Clinic-based | 9 | .20 | -.02, .42 |  |  |  |
| Non-clinic-based | 4 | -.09 | -.42, .24 |  |  |  |
| MI focus |  |  |  | 2.96 | .095 |  |
| Both PA and diet | 10 | .18 | -.04, .40 |  |  |  |
| Diet | 3 | -.27 | -.75, .20 |  |  |  |
| Parent involvement |  |  |  | .11 | .737 |  |
| Yes | 5 | .06 | -.24, .37 |  |  |  |
| No | 8 | .13 | -.13, .39 |  |  |  |
| Intervenor |  |  |  | .51 | .474 |  |
| PCP | 6 | .18 | -.14, .50 |  |  |  |
| Non-PCP | 7 | .02 | -.28, .32 |  |  |  |
| Booster |  |  |  | 4.98 | **.026** |  |
| Yes | 8 | -.06 | -.29, .18 |  |  |  |
| No | 5 | .37 | .08, .65 |  |  |  |
| As an add-on |  |  |  | 3.45 | .063 |  |
| Yes | 6 | -.12 | -.42, .19 |  |  |  |
| No | 7 | .27 | .002, .54 |  |  |  |
| Fidelity check |  |  |  | 6.01 | **.014** |  |
| Yes | 7 | .29 | .07, .52 |  |  |  |
| No | 6 | -.15 | -.43, .12 |  |  |  |

**Supplemental Table 3**. Moderators for the effects on reducing snack intake (*k* = 10)

| **Continuous moderators** | **k** | **Slope B** | **SE** | **Tau^2^** | **Q_between_** | **p-value** |
| --- | --- | --- | --- | --- | --- | --- |
| Intervention duration | 10 | -.007 | .02 | .008 | .12 | .724 |
| MI sessions | 10 | -.04 | .02 | 0 | 6.58 | **.010** |
| **Categorical moderators** | **k** | **Effect size** | **95% CI** | **Q_between_** | **p-value** |  |
| Age |  |  |  | 2.52 | .113 |  |
| < 10 years | 7 | -.13 | -.29, .03 |  |  |  |
| ≥ 11 years | 3 | -.33 | -.52, -.14 |  |  |  |
| Baseline weight |  |  |  | .05 | .819 |  |
| Non obese | 5 | -.20 | -.41, .01 |  |  |  |
| Obese | 5 | -.23 | -.42, -.05 |  |  |  |
| Purpose |  |  |  | 2.04 | .153 |  |
| Prevention | 8 | -.26 | -.40, -.12 |  |  |  |
| Treatment | 2 | 0 | -.32, .32 |  |  |  |
| Setting |  |  |  | .12 | .731 |  |
| Clinic-based | 6 | -.24 | -.44, -.05 |  |  |  |
| Non-clinic-based | 4 | -.19 | -.40, .01 |  |  |  |
| MI focus |  |  |  | .14 | .708 |  |
| Both PA and diet | 7 | -.21 | -.37, -.05 |  |  |  |
| Diet | 3 | -.28 | -.60, .05 |  |  |  |
| Parent involvement |  |  |  | 2.80 | .094 |  |
| Yes | 9 | -.18 | -.30, -.05 |  |  |  |
| No | 1 | -.49 | -.83, -.14 |  |  |  |
| Intervenor |  |  |  | .79 | .373 |  |
| PCP | 5 | -.29 | -.50, -.08 |  |  |  |
| Non-PCP | 5 | -.16 | -.35, .02 |  |  |  |
| Booster |  |  |  | 5.20 | **.023** |  |
| Yes | 3 | .01 | -.21, .22 |  |  |  |
| No | 7 | -.28 | -.41, -.16 |  |  |  |
| As an add-on |  |  |  | .83 | .362 |  |
| Yes | 4 | -.15 | -.36, .06 |  |  |  |
| No | 6 | -.28 | -.47, -.09 |  |  |  |
| Fidelity check |  |  |  | 1.33 | .249 |  |
| Yes | 3 | -.11 | -.34, .12 |  |  |  |
| No | 7 | -.27 | -.43, -.11 |  |  |  |

**Supplemental Table 4**. Moderators for the effects on increasing dairy intake (*k* = 10)

| **Continuous moderators** | **k** | **Slope B** | **SE** | **Tau^2^** | **Q_between_** | **p-value** |
| --- | --- | --- | --- | --- | --- | --- |
| Intervention duration | 10 | -.02 | .02 | 0 | 1.19 | .276 |
| MI sessions | 10 | -.03 | .02 | 0 | 3.06 | .080 |
| **Categorical moderators** | **k** | **Effect size** | **95% CI** | **Q_between_** | **p-value** |  |
| Age |  |  |  | .22 | .639 |  |
| < 10 years | 6 | -.01 | -.22, .19 |  |  |  |
| ≥ 11 years | 4 | .05 | -.14, .25 |  |  |  |
| Baseline weight |  |  |  | .04 | .835 |  |
| Non obese | 6 | .04 | -.16, .23 |  |  |  |
| Obese | 4 | .01 | -.20, .21 |  |  |  |
| Purpose |  |  |  | .25 | .617 |  |
| Prevention | 9 | .01 | -.14, .16 |  |  |  |
| Treatment | 1 | .14 | -.35, .63 |  |  |  |
| Setting |  |  |  | 4.90 | **.027** |  |
| Clinic-based | 6 | .12 | -.01, .26 |  |  |  |
| Non-clinic-based | 4 | -.14 | -.34, .05 |  |  |  |
| MI focus |  |  |  | .50 | .479 |  |
| Both PA and diet | 8 | -.002 | -.16, .15 |  |  |  |
| Diet or PA | 2 | .13 | -.19, .44 |  |  |  |
| Parent involvement |  |  |  | .50 | .479 |  |
| Yes | 6 | -.02 | -.21, .16 |  |  |  |
| No | 4 | .08 | -.14, .30 |  |  |  |
| Intervenor |  |  |  | 2.37 | .124 |  |
| PCP | 4 | .13 | -.05, .30 |  |  |  |
| Non-PCP | 6 | -.07 | -.25, .11 |  |  |  |
| Booster |  |  |  | 5.19 | **.023** |  |
| Yes | 5 | .17 | .01, .33 |  |  |  |
| No | 5 | -.08 | -.23, .07 |  |  |  |
| As an add-on |  |  |  | 4.48 | **.034** |  |
| Yes | 8 | .09 | -.03, .22 |  |  |  |
| No | 2 | -.21 | -.47, .04 |  |  |  |
| Fidelity check |  |  |  | .58 | .447 |  |
| Yes | 4 | .09 | -.13, .32 |  |  |  |
| No | 6 | -.02 | -.19, .16 |  |  |  |

**Supplemental Table 5**. Moderators for the effects on decreasing sugary beverages (*k* = 21)

| **Continuous moderators** | **k** | **Slope B** | **SE** | **Tau^2^** | **Q_between_** | **p-value** |
| --- | --- | --- | --- | --- | --- | --- |
| Intervention duration | 21 | -.003 | .006 | .11 | .18 | .667 |
| MI sessions | 21 | -.06 | .03 | .07 | 4.88 | **.027** |
| **Categorical moderators** | **k** | **Effect size** | **95% CI** | **Q_between_** | **p-value** |  |
| Age |  |  |  | 1.74 | .187 |  |
| < 10 years | 15 | -.09 | -.28, .10 |  |  |  |
| ≥ 11 years | 6 | -.33 | -.63, -.03 |  |  |  |
| Baseline weight |  |  |  | 1.53 | .217 |  |
| Non obese | 13 | -.13 | -.50, .24 |  |  |  |
| Obese | 9 | -.50 | -.95, -.05 |  |  |  |
| Purpose |  |  |  | .57 | .451 |  |
| Prevention | 18 | -.14 | -.32, .04 |  |  |  |
| Treatment | 3 | -.33 | -.78, .13 |  |  |  |
| Setting |  |  |  | .58 | .446 |  |
| Clinic-based | 15 | -.36 | -.75, .02 |  |  |  |
| Non-clinic-based | 7 | -.10 | -.66, .45 |  |  |  |
| MI focus |  |  |  | .26 | .607 |  |
| Both PA and diet | 18 | -.24 | -.60, .12 |  |  |  |
| Diet or PA | 4 | -.47 | -1.25, .32 |  |  |  |
| Parent involvement |  |  |  | .27 | .605 |  |
| Yes | 16 | -.33 | -.72, .05 |  |  |  |
| No | 6 | -.14 | -.76, .48 |  |  |  |
| Intervenor |  |  |  | .47 | .492 |  |
| PCP | 10 | -.41 | -.90, .09 |  |  |  |
| Non-PCP | 12 | -.17 | -.62, .28 |  |  |  |
| Booster |  |  |  | .78 | .377 |  |
| Yes | 6 | -.05 | -.35, .26 |  |  |  |
| No | 15 | -.21 | -.41, -.01 |  |  |  |
| As an add-on |  |  |  | .55 | .459 |  |
| Yes | 11 | -.22 | -.45, .01 |  |  |  |
| No | 10 | -.09 | -.34, .16 |  |  |  |
| Fidelity check |  |  |  | 2.50 | .114 |  |
| Yes | 10 | -.05 | -.26, .17 |  |  |  |
| No | 11 | -.29 | -.51, -.07 |  |  |  |

**Supplemental Table 6**. Moderators for the effects on increasing MVPA (*k* = 24)

| **Continuous moderators** | **k** | **Slope B** | **SE** | **Tau^2^** | **Q_between_** | **p-value** |
| --- | --- | --- | --- | --- | --- | --- |
| Intervention duration | 24 | -.006 | .005 | .08 | 1.16 | .281 |
| MI sessions | 24 | .03 | .02 | .08 | 1.61 | .205 |
| **Categorical moderators** | **k** | **Effect size** | **95% CI** | **Q_between_** | **p-value** |  |
| Age |  |  |  | 5.89 | **.015** |  |
| < 10 years | 8 | .01 | -.20, .22 |  |  |  |
| ≥ 11 years | 16 | .35 | .18, .52 |  |  |  |
| Baseline weight |  |  |  | 4.27 | **.039** |  |
| Non obese | 9 | .08 | -.11, .26 |  |  |  |
| Obese | 15 | .34 | .17, .52 |  |  |  |
| Purpose |  |  |  | .44 | .509 |  |
| Prevention | 18 | .20 | .05, .34 |  |  |  |
| Treatment | 6 | .31 | .001, .62 |  |  |  |
| Setting |  |  |  | .14 | .710 |  |
| Clinic-based | 16 | .20 | .03, .37 |  |  |  |
| Non-clinic-based | 8 | .26 | .01, .50 |  |  |  |
| MI focus |  |  |  | 1.19 | .276 |  |
| Both PA and diet | 17 | .17 | .01, .34 |  |  |  |
| PA | 7 | .35 | .08, .63 |  |  |  |
| Parent involvement |  |  |  | 2.55 | .110 |  |
| Yes | 12 | .11 | -.08, .30 |  |  |  |
| No | 12 | .34 | .14, .54 |  |  |  |
| Intervenor |  |  |  | .12 | .727 |  |
| PCP | 9 | .25 | .03, .46 |  |  |  |
| Non-PCP | 15 | .20 | .02, .37 |  |  |  |
| Booster |  |  |  | .25 | .615 |  |
| Yes | 11 | .26 | .05, .47 |  |  |  |
| No | 13 | .19 | .002, .37 |  |  |  |
| As an add-on |  |  |  | .001 | .972 |  |
| Yes | 12 | .22 | .03, .40 |  |  |  |
| No | 12 | .22 | .02, .42 |  |  |  |
| Fidelity check |  |  |  | .09 | .759 |  |
| Yes | 15 | .20 | .03, .37 |  |  |  |
| No | 9 | .25 | .03, .46 |  |  |  |
| PA measurement |  |  |  | 4.21 | .122 |  |
| Accelerometer | 7 | .36 | .12, .60 |  |  |  |
| Pedometer | 2 | .62 | -.02, 1.27 |  |  |  |
| Self/proxy-report | 15 | .12 | -.05, .29 |  |  |  |

**Supplemental Table 7**. Moderators for the effects on decreasing screen and sedentary time (*k* = 16)

| **Continuous moderators** | **k** | **Slope B** | **SE** | **Tau^2^** | **Q_between_** | **p-value** |
| --- | --- | --- | --- | --- | --- | --- |
| Intervention duration | 16 | -.007 | .002 | 0 | 9.91 | **.002** |
| MI sessions | 16 | -.03 | .02 | .002 | 2.90 | .089 |
| **Categorical moderators** | **k** | **Effect size** | **95% CI** | **Q_between_** | **p-value** |  |
| Age |  |  |  | .59 | .444 |  |
| < 10 years | 10 | -.08 | -.18, .02 |  |  |  |
| ≥ 11 years | 6 | -.01 | -.16, .14 |  |  |  |
| Baseline weight |  |  |  | .31 | .581 |  |
| Non obese | 12 | -.05 | -.15, .05 |  |  |  |
| Obese | 4 | -.11 | -.32, .10 |  |  |  |
| Purpose |  |  |  | .81 | .367 |  |
| Prevention | 12 | -.04 | -.14, .05 |  |  |  |
| Treatment | 4 | -.15 | -.37, .07 |  |  |  |
| Setting |  |  |  | 3.36 | .067 |  |
| Clinic-based | 11 | -.11 | -.20, -.01 |  |  |  |
| Non-clinic-based | 5 | .05 | -.09, .19 |  |  |  |
| Parent involvement |  |  |  | 1.37 | .242 |  |
| Yes | 10 | -.09 | -.19, .01 |  |  |  |
| No | 6 | .01 | -.13, .15 |  |  |  |
| Intervenor |  |  |  | .91 | .341 |  |
| PCP | 7 | -.10 | -.22, .02 |  |  |  |
| Non-PCP | 9 | -.02 | -.14, .10 |  |  |  |
| Booster |  |  |  | .05 | .827 |  |
| Yes | 6 | -.08 | -.24, .09 |  |  |  |
| No | 10 | -.05 | -.16, .05 |  |  |  |
| As an add-on |  |  |  | 1.83 | .176 |  |
| Yes | 8 | -.004 | -.12, .11 |  |  |  |
| No | 8 | -.12 | -.23, .002 |  |  |  |
| Fidelity check |  |  |  | .69 | .405 |  |
| Yes | 13 | -.08 | -.17, .02 |  |  |  |
| No | 3 | .01 | -.17, .20 |  |  |  |
